# Observations on Certain Cases of Secondary Disease, Subsequent to the Measles, in a Letter to Benjamin Rush, M. D.

**Published:** 1808-02

**Authors:** 


### *Observations on certain Cases of secondary Disease, subsequent to the Measles, in a Letter to* Benjamin Rush, M.D. *by* ———.

My dear Sir,

Lest you should either not have seen it, or have forgotten it, I am desirous to put into your hands a short Treatise on the Measles of Jamaica, which the author represents (and I think truly), as differing from the usual Measles of Europe. But before I proceed, I wish to make known to you certain cases (as I apprehend them to be), of secondary disease; which followed a visitation of the measles, in 1802, in the township where I reside, which lies about N. lat. 44° 16′, and is forty miles from the sea.

This visitation appeared with us in the early summer months. As usual in this complaint, some of the patients called for medical advisers, some managed for themselves, and some let the disorder take its course. Many patients, in the space of some weeks or months after their attack had ceased, experienced considerable illness; which, or at least the particular form of which, I am inclined to attribute to the influence of measles, which had been ill cured.—From the cases of this description, I have selected five of the dysenteric class, which occurred under my own notice, in two families living close to each other, in a healthy part of the country; and of which I can speak with some certainty, as three of them ended fatally, and were followed, at my desire and in my presence, by dissections. I add a sixth case of the dysenteric kind, which took place within my own knowledge also, but under mixed circumstances; that it may serve in our general comparison. Two other cases will afterwards be touched upon, of a pulmonic nature, which followed ill-managed measles; and seemed to have a great correspondence with the malady of the five first of our patients.

### CASES.

Case I. August 9, 1802, in the afternoon, a healthy little girl, nearly three years old, was seized with some dysenteric symptoms. The next afternoon she had fits, which left her insensible; but in the evening, her senses returning, she rose and walked into an adjoining room. The stools which still occurred continued loose, and of various colours and kinds; but she complained of no pain, except in her bowels; and even there, the pain seemed rather partaking of distress, than violent or constant. On the 13th, her pulse became exceedingly slender; her hands grew cold before the rest of her body, and at midnight she quietly expired.

August 14, the body was opened. The larger intestines, from the cæcum to the rectum, appeared inflamed in their inside, under the villous coat. They were also much thickened, and the outer coat was so hardened, as to appear to have advanced a step towards becoming cartilaginous; exhibiting, as to mass and substance, some faint resemblance to the gizzard of a young fowl.—On the mesocolon, and on the outside of the larger intestines, were oval oval tubercles, to the number of 80 or 100. Some were of the size of a dwarf (or French) bean; their colour was a dark red, and their consistence equal to that of the liver; but they were free from ulceration. The mesentery had many of its glands enlarged, or at least it exhibited many tubercles; of which some were inflamed. One portion of the smaller intestines also was inflamed to a considerable extent, but it had no tubercles.

The intestinal canal, in various parts, contained soft fæces; but had neither hard balls of fæces, nor contractions, nor one part of the intestines thrust within another part; nor could any other unusual affection be perceived in them.

One worm (a *lumbricus teres*), was discovered with faint remains of life. But no perforation of the gut was seen in the parts examined, nor any worm in the cavity of the abdomen, which could explain the fit.

A part of the left lobe of the liver had its blood-vessels turgid; and the liver, in general, seemed much enlarged. The gall bladder was full of dark-coloured bile.

The outer integument of the right kidney was inflamed.

The lungs, as seen through one part of the diaphragm, appeared to be sound.

Case II. August 14. A boy, about two years and a half old, living in the next house, and apparently of an excellent constitution, was seized much in the same manner; that is, with violent purging, attended with fever, blood, mucus, and tenesmus. The fever ran high, but the pain by no means appeared to be considerable. He died on the 17th, in a fit.

On the 18th, the body was opened. The minutes of this case being lost, I can only state, in general, that the tubercles which were here discovered, were diffused over the upper, as well as over the lower intestine, and still retained their station on the mesentery and mesocolon; their number amounting in the whole, perhaps, to 200. There were worms also, like the preceding, in the intestinal tube; but they were here 22 in number, and of the medium length, perhaps, of eight inches.

Case III. August 15. A female infant was violently seized with dysenteric symptoms, attended, however, with no remarkable degree of pain. After a struggle of five or six weeks, she recovered.

CASE IV. August 17. A girl, aged six years and eight months, was taken ill with dysenteric symptoms; but, like the rest, she experienced little pain, being able to move about the room till a short time before she died; which happened on the 29th.

Her body being opened, the tubercles were found scattered, as in the second case; but they prevailed most on the outer surface of the greater intestines. Some of those tubercles had either receded from an inflammatory state, or had not advanced into it; for their colour was yellow. Some were as large as a kidney (or English) bean. Some had penetrated, so as to shew themselves on the inner surface of the gut. One tubercle had separated, owing to the mortification of the surrounding parts; and this separation was on the inner side of the intestine. The whole of the colon was inflamed; it was also thickened, but it was tender to the feel; and within, it was of a dark red colour, resembling that of a ripe mulberry; being under the operation of gangrene.

Worms, like the preceding, appeared to the number of 24; some of which had entered the œsophagus.

The liver exhibited little that was peculiar.

The spleen was unusually soft and livid.

The integuments of the kidney were not noticed.

The lungs, as seen through the diaphragm, appeared in a natural state.

CASE V. August 18. Another girl, whose age somewhat exceeded five years, was taken ill in a similar manner, and retained the symptoms for several weeks; but finally recovered, and is still a healthy child.

None of these children (for it will be observed that these were all children), I say not one of them was attended by a practitioner, during their attack with measles; as they lived in the country, and applied for no assistance.

CASE VI. About the same time, another female infant, for whose original attack of the measles some Glauber's salts had been given, though in an advanced state of them, was seized with dysenteric symptoms; but she was soon relieved by new doses of Glauber's salts. This infant, thus imperfectly attended in the measles, resided in a thick settled village.

The two other cases, which were of a pulmonic nature, and succeeded measles, will be spoken of hereafter.—

These

These two patients were young men, about twenty years of age.

I shall conclude, what respects the statement of these cases, by three general remarks.—1st. I do not mention all the instances of disease which arose at this period, in our neighbourhood, after ill-cured measles; because I cannot, at present, be equally certain of the particulars, as in the instances above recited.—2d. I may affirm, that none of those who were gently and early evacuated and bled, experienced a severe attack of measles; or any subsequent disease, which could be ascribed to measles.—3d. No tendency, in general, to dysentery, manifested itself at this time within our little circle; and, as to pulmonary complaints of a chronic nature, they are at all times somewhat uncommon among us. The two last of these remarks appear to me of considerable importance, as to the question before us.

The first five cases recited above, I may now observe, seem to give room for the following *queries*.

*Query* 1. Had poison, whether arising from wild plants or other articles, accidentally eaten, any concern in this business?

The answer is easy in the instances before us. Two of the children, mentioned in the first six cases, were infants at the breast; and one of them, for the most part, lived separate from the others. The other cases, also, to which I afterwards referred, were accompanied with circumstances, rendering this supposition too improbable for further notice.

*Query* 2. If measles, and especially ill-cured measles, in some cases and some countries, are said to lead to tubercles in the lungs; why may not tubercles on the intestinal tube and its connected membranes, follow, also, from the same sources in other cases and in other countries?

As this query seems reasonable, it will pass only with this comment. It is by means of these tubercles, that we may possibly point out a correspondence between the pulmonic and dysenteric cases above referred to; a correspondence, which will receive further evidence, from the circumstance of the same treatment having appeared to succeed in the cure of each.

*Query* 3. If we say that the above patients, whose bowels were affected, had pure and independent dysentery, how shall we reply to the following difficulties?

1st. Dysenteric symptoms (generally speaking), never occurred with us, as I have mentioned, at this period, unless less in cases where measles were concerned. Secondly, in various parts of the world, fluxes often happen in the primary state of measles; as Sydenham, Huxham, Watson, Cullen, and others, have witnessed in Great Britain. Tissot, also has noticed these fluxes in Switzerland; Rhazes (as quoted by Mead) speaks of them in the East; and the author (soon to be mentioned) found them to be a fixed part of the disease in Jamaica. In our neighbourhood, the only variation is, that these dysenteric symptoms occurred after a little interval, and not in immediate and visible connection with the measles. Thirdly, the mortality with us was greater and more rapid, than in common dysenteric cases, where measles have no concern. Fourthly, I am inclined to believe that in proper dysentery, the tubercles which are sometimes found in dissecting the intestines, shew themselves generally within the intestinal tube in the first instance; and not without it, as with our patients. Fifthly, remedies which are not usually employed in dysentery, seem to have had influence, as we shall soon find, over three of our bowel-cases which were desperate. It is true, that we must abate somewhat from this last argument: since it may be thought that the common treatment for malignant dysentery is as yet imperfect; and that the very medicines employed in this case may themselves be of use in future trials with dysentery. Such are the reasons to be urged in favour of the disease having been only a certain kind of dysentery, specially connected in certain situations with measles.

But it is to be urged, on the other hand, that in one house, where three of the children had been sick, three men were seized with apparently true dysenteries, while attending them; and another man was seized with the same complaint, who occasionally attended two others of the sick children in a neighbouring house: and it may be added, that if pure phthisis and pure ophthalmia may follow measles, as their cause; so, possibly, may pure dysentery.

Perhaps then we may find reason to decide thus between the two opinions. Measles may have left a ready disposition in the children to receive true dysentery from the common causes of dysentery; and when dysentery has once occurred, it may have exasperated that tendency to tubercles, which every where seems to be a frequent attendant upon measles.

*Query* 4. Even upon the supposition that measles had their share (direct or indirect) in the above diseases; it may may still be inquired, whether the tubercles which were discovered in the abdomen, preceded, or whether they only accompanied or followed, the new state of inflammation?—In other words, since the pain at the commencement of tubercles or schirrus, is often so small, as not to be noticed; we may ask, whether the tubercles in question existed unobserved, before the occurrence of the new disease? or did they (see cases 1 and 2) begin and ripen within 108 hours, or some shorter period, before death?—The same question attends the thickening and hardening of the intestines.

The reasonableness of the suggestions in this query as to the sudden rise of tubercles, especially where there was a previous tendency to them, may be made manifest by a statement of two or three facts.

A sudden swelling of the glands lying within our sight or feeling, is seen in sore throats; and again it is seen in irritations of the lymphatic system from venereal buboes, and other causes both natural and artificial: and the disappearance of many such swellings is often as abrupt as their appearance. As to the internal examples of sudden swellings of glands or tubercles, take the following produced by Dr. John Hume, formerly a commissioner for the sick and hurt seamen of the British navy; who writes as follows: "Dr. Mackittrick Adair, (of Antigua,) who had practised physic for a number of years in the West Indies, mentioned his having opened a man who died the third day of the yellow fever. The whole body, he says, had somewhat of a livid appearance; the cavity of the abdomen seemed dry and destitute of serum; the omentum was full of black blood, and had on its lower edge a number of round glandular bodies full of the same sort of black blood; the coats of the stomach, duodenum, and ilium, were remarkably inflamed; the liver was not increased in its bulk, though its texture seemed vitiated; the gall bladder was full of black bile; and there were some round worms in the cavity of the intestines; the urinary bladder was a little inflamed; the lungs were sound; and the pericardium contained a more viscid yellow serum, and in larger quantities than common; and the cellular membranes were every where filled with a tough clay-coloured mucilage."—Thus then we have discovered round glandular bodies, filled with black blood, on the cawl of a man who died on the third day of a fever; that is, before it had raged 72 hours. In this particular instance also, the viscid serum and tough mucilage may go some way towards wards explaining the more immediate cause of these glandular bodies.

Tubercles have been found repeatedly in the bowels of patients, who have died of dysentery; but they are more frequent in some circumstances or situations than in others: and in some of the dissections made, the thickening, the hardening, and the subsequent softening (by mortification) of the intestines, have also been particularly noticed. Some of the cases thus described, had a speedy termination in death, (though less speedy than with us;) but others of the cases had some duration.*

I shall finish what regards the description of our cases, compared with those of the serious epidemic dysentery, by observing that where the seat of the tubercles in epidemic dysentery is particularly noticed, it is said to be within the intestines; and not without, as with us. It is also observable, that the patients examined by others were adults; whereas ours, who had bowel-affections, were children; who (as is well known) are peculiarly liable to swellings of the mesenteric glands.—The pulmonic complaints also fell by preference, as was natural, on those arrived at the age of puberty.

Let us now speak of what was done for the relief of our patients.

### *Treatment of the above Cases.*

The first of our six cases having in its commencement appeared to belong to proper dysentery, medicines were administered accordingly. But the mother of this patient quickly

* Sir John Pringle has given his dissections in dysentery more at large perhaps than any other author. They were of adult patients, male and female. (See Army Diseases, 7th Edition, p. 237 to 250.)—Dr. Baillie, not finding many cases of tubercles in the intestines in Great Britain, refers chiefly to Pringle. (See Morbid Anatomy, 2nd Edition, p. 174-175.)—Sir Gilbert Blane, who superintended several dissections in the West Indies, yet refers chiefly to the same author. (See Diseases of Seamen, 3rd Edition, p. 450-453.)—Dr. Donald Monro, thought that the tubercles discovered by Sir John Pringle and Sir G. Baker in their dissections, were uncommon; since in old dysenteries, he himself had seen little besides spots and erosions; appearances, we may add, which with small exceptions, were confined to the inner parts of the larger intestines. (See Edinburgh Essays and Observations, Literary and Physical, 3. 516-524; where he gives particulars, which he had refrained from inserting in his work on Military Hospitals.) He had once observed the mesenteric glands ossified, though not by dysentery. (London Medical Transactions, 2. 362-365.—I cannot refer to Dr. Stark; my copy of his work being at present unfortunately missing.

quickly objecting to every thing tending to nausea, and worms being suspected to have some share in the fit; small doses of calomel were alone continued; symptoms in other respects being combated as they arose.—The lancet was once employed in the second case; and attempts also were made to obviate inflammation and obstruction, without forgetting the dysenteric symptoms (in the common acceptation of that term.)

The surviving patients, however, were still going on unfavourably, when I recollected the little treatise on the measles in Jamaica, by Mr. Quier; to which I alluded in the beginning of this letter. With the consent of a medical friend whom I had a little before called in, the following medicines were adopted out of it, and given every six hours; namely, castor oil, weakened with a proportion of olive oil; magnesia, or the testaceous powders, employed discretionally according to the state of the body, but commonly alternately; gum arabic; and an opiate. Clysters also were given on the same plan.—I forget what share calomel had in our prescriptions. The result, however, was as follows. The third and fifth patients escaped; and the life of the fourth was prolonged under desperate circumstances. For the fifth patient, the lancet was once used; but I shall perhaps be blamed for not having advised a very liberal employment of it in the whole of the four cases which succeeded the first dissection; and especially in the beginning of them. Sydenham indeed had advised it in cases of flux accompanying measles; but our ideas were not yet sufficiently firm as to the connection between the present complaint and measles; and the fear of the public opinion (where several deaths had occurred) was too considerable, to allow us to vary much; and especially in an ostensible manner, from the common practice for the cure of dysentery.

The sixth case, standing on separate grounds, furnishes no direct evidence as to the new treatment; but only presents a contrast to the other cases, whether it be considered as proper dysentery, or as a less exasperated state of secondary disease.

It is not only on the cases which are related above, that I have founded my conjecture as to the utility of the foregoing medicines, when thus combined. For I employed them in the two cases where pulmonic affections occurred, subsequent to ill-cured measles; though in one of these cases, the appearances had almost precluded hope. A diet of milk and vegetables was at the same time recommended; mended; but no other medicines concurred in the cure, unless perhaps antimonials and elixir of vitriol, and yet the recovery seemed more regular and comparatively more speedy, than in the cases where the intestines alone had suffered.

I am the more inclined to make further experiment of the above method of treatment in future cases, from observing that Dr. Watson (senior) says, that with patients in the worst kind of measles, "a draught of fresh milk, either alone or mixed with water, was most grateful, and tended much to the alleviation of their complaints, as well as recruiting their strength." Sydenham among his remedies, recommended weak milk and water boiled, and demulcents; milk, demulcents, and an oily linctus made part of the treatment of Mead. Dr. Percival used a demulcent and a neutral salt in his prescriptions; to say nothing of other authorities.

In mentioning these things, we do not mean to reject other aids; as will be seen in particular, when we speak of the diarrhœa in measles.

But to return to the oil, let us ask whether oil may not be useful in some shape or other, in cases of tubercle, and even of schirrus, viewed in a more extensive sense?—Olive oil, simmered away very gradually to the consistency of an ointment, and applied externally to cancer (as recommended in some of our newspapers) is said to have had a happy influence over an open devouring cancer in this neighbourhood. May not oily medicines, aided by the above named accompaniments, (and in particular by the mucilage of the gum and the removal of costiveness,) have their turn of trial in an internal form, in the case of cancer? And should not many of those cancer-medicines, which in modern trials have uniformly failed, cease to be longer administered; as being proved, in our hands, to be of all others the most hopeless in general cases?—Should difficulty occur in making the oils sit easy upon certain stomachs, perhaps spermaceti may admit of being used in their stead: though it may be doubted in general how far an increase of the quantity of meat, broth, butter, and other such animal substances, may be useful in cases of schirrus and cancer. What would be the effect of soap, which is composed of oil and an alkali, in case it should be sufficiently mild, may deserve a question.

Of

### *Of Measles generally considered.*

I think it may safely be observed, that there are few instances of diseases like the measles, which are of such magnitude when neglected; and yet are commonly to be checked by such light measures in the commencement. A single bleeding and a single purge early administered, and repeated in case of need, with low diet and pure and temperate air, have, with little or no exception, sufficed in our climate and our situation, for the common form of the first disease; but (according to our experience in 1802) it prevents what are far more to be dreaded, namely, the secondary complaints. As a purge and a bleeding then cannot injure a common constitution, neither of them ought ever to be spared, as far as the cure demands them; which I believe will commonly be found to be within a small extent. Some give a vomit on the first occurrence of measles; and it perhaps may be useful in various points of view; but I have never seen this method extensively practised; and some have even objected to it.

I shall not pretend to teach at large of the nature of measles, I shall be content with asserting, that at first, the symptoms seem to be inflammatory; and that in violent cases they go on to indicate acrimony; insomuch that corrosion often follows the lancet and blister, and (if I remember right) the scratch and even the pustule.

I think we ought not to be so confident, as Dr. Watson appears to have been, that measles of the malignant kind are putrid, otherwise than as the result of acrimony; though his recommendation of the Peruvian bark for removing debility, where the chest is free, may at first sight seem a proof in his favour. I believe, however, that his observation is thoroughly just, that purulent collections of matter do not occur even in the worst shape of measles. In their more malignant form, every thing tends to corrosion of the flesh, to caries of the adjoining bones, and to ultimate general mortification.

Dr. Percival, in the spring and summer of 1774, found the measles of the regular kind epidemic at Manchester, and gives the following particulars respecting them. He says, that "it was not unusual for violent peripneumonic symptoms to occur five, six, or even eight days after the disappearance of the eruption. Under these circumstances, bleeding, blisters, and the seneka root, were found to be very efficacious remedies. I prescribed (he adds) the Peruvian bark with great success to many of my patients under under the measles, combining it with demulcents and the saline mixture; and premising venesection, when the signs of inflammation were urgent. The practice of giving bark in this disease (he affirms) was first introduced by Dr. Cameron, a very eminent physician at Worcester; who observed, that it prevents the retrocession of the morbid acrimony, and continues the efflorescence on the skin, sometimes so long as the twelfth day. By this salutary operation the cough and other inflammatory symptoms are in a great measure obviated; and the patient is freed from all danger of a peripneumony, the fatality of which Sydenham describes in such strong terms. It is many years (continues Dr. Percival) since I first adopted the method of cure recommended by Dr. Cameron; and experience has afforded me the fullest conviction of its safety and efficacy, in all ordinary cases. During the late epidemic, not a single instance occurred to me of the peripneumony succeeding the measles, when the bark had been employed: but my assistance was desired in the last stage of fifteen unfortunate cases of this kind, in which the common antiphlogistic and pectoral course had been pursued. The measles, when violent in degree or ill treated, frequently lay the foundation of hectic fevers or pulmonic consumptions*."

Of course we cannot overlook in this account the mention of a demulcent (namely liquorice,) as well as of the neutral salts.—But what is suggested concerning the Peruvian bark is of more immediate interest. I do not mean as to the mere history of its application to cases of this sort; for here Dr. Percival is mistaken. At the visitation of the measles in Edinburgh, in 1735 and 1736, it is said, that "to those who seemed to be hectic, and to be threatened with a phthisis after this disease; vomits, Peruvian bark, and asses milk, were of service†." Dr. Whytt also employed

* Medical Observations and Inquiries, London, 5. 283-285. Edition 1776.

† Medical Essays and Observations, Edinburgh, 5. 28. Fifth edition.—In the same volume, p. 84, we find a paper by the elder Dr. Monro, on the effect of the Peruvian bark in gangrenes, agues, and small pox; which perhaps shows by what steps the bark first came to be applied to measles; namely, in order to fill the pustules, improve the matter in them, and prevent mortification.

With the same view, Haller, in the case of black pustules in the small pox, ordered camphor; and his relation of its success, even when the urine had become green, forms the most interesting article in the Pathological Observations employed the bark for hoarseness after measles, in 1757, of which we shall soon say more; and Dr. Watson used it more at large in this disease in 1763. But I think you will agree, that it is more useful to notice the supposed power of bark over the eruption, and the supposed power of the eruption over the morbid acrimony, than any other circumstance in the account by Dr. Percival. This, if correctly stated and generally confirmed, may lead us to one mode of controlling both the primary and secondary diseases of measles. It even induces us to ask, in case the morbid matter can be made to expand itself in some degree by means of the eruption; whether severe cases of the primary disease, which happen in spite of proper treatment, may not occasion the secondary diseases to be less formidable than might otherwise be expected? As also, whether this insidious distemper is not sometimes more particularly to be feared in its milder forms, if neglected or mismanaged. It is next in order to mention again, that Dr. Whytt found it advantageous to use the bark for the hoarseness sometimes succeeding measles; whether attended with cough, or otherwise. It was combined with vinegar, though probably without necessity*. The bark, joined with tincture of cantharides and opiates, has likewise often been found serviceable in the hooping cough; as opiates with bark and chalybeates, have also been employed successfully in other coughs. Of course the universal rule for all these cases is, to get rid, in as great degree as possible, of the more formidable symptoms of inflammation, before the bark is admitted. The favourable operation of the bark here, is probably not so much owing to its specific powers, as to its stimulating the powers of the constitution, or to its creating counter-actions sufficient to balance the morbid powers of the disease. I must not quit the subject of the bark, without mentioning that Dr. Whytt used the bark for dysentery attended with aphthæ; though he agreed with Cleghorn, that it did no service where mortification had commenced †. This new application of bark has full relation to our general subject; not vations of this author. (See them translated into English, and printed in London for Wilson and Durham, in 1756.) Dr. Cullen supposes the chief advantage of camphor, in confluent small pox, to arise from its antiseptic qualities.

* Essays and Observations, Physical and Literary, Edinburgh, vol. 3. Art. 14. Edition 1771.

† Essays and Observations, Physical and Literary; as last cited.

only because dysenteric symptoms, but (as we shall hereafter find) aphthæ, are frequently combined with measles. After all, the best mode of treating measles may perhaps be one, which shall have little to do with bark. Several plans in medicine have in their day met with applause, merely because they were somewhat preferable to those which went before: and so it may possibly be with those which include the exhibition of bark in measles. The subject therefore merits new attentions. Here, however, I shall leave these particulars, to make another reference to Dr. Percival.

This amiable author (now no more) relates from the records of Manchester, in England, that in a period of six years, measles (contrary to small-pox) were found most fatal to males. He says also, that in this period, the measles destroyed a number equal to about one sixth part of the number destroyed by the small-pox; and that one half of this loss occurred with patients under two years of age*. With respect to the numbers lost however, the Manchester records are probably deceitful; a part of the slaughter occasioned by measles having been concealed under other names. If we believe Morton, this disease has sometimes become a little plague in its more ostensible shape; and when comprehended under all its forms (primary and secondary) it is truly a formidable scourge. Sydenham says of the cough, " after the measles go off, that it is so very fatal, that it may well be reckoned the chief minister of death; destroying even more than the small pox †." Happily some of these accounts surpass modern experience, at least in the United States and in Great Britain.

For the cure of the diarrhœa in measles, Sydenham (as I have suggested above) chiefly relied on the lancet; which, (as is well known) he boldly used with every patient in every stage of measles which seemed to require it; though in common measles, he says, that he generally was able by his methods to proceed without bleeding, in the first stages; an assertion to which we cannot refuse a certain degree of credit. At Edinburgh, in 1735 and 1736, " if there was a diarrhœa (as we are told,) blood-letting, vomits, and the decoctum album generally put it away ‡.

Doctor

---

* Medical Observations and Inquiries, vol. 5, p. 283, 288, 289.

† See Peachy's Translation, p. 135, 5th Edition; which happens now to lie before me.

‡ See Medical Essays and Observations as above, 5. 27. The decoctum album

Doctor Cullen will afford me some short concluding quotations on the subject of measles.—He says, that "a diarrhœa frequently comes on (after the desquamation of the eruption,) and continues for some time;" also, that "it is common for the measles when they have been of an inflammatory kind, to be followed by inflammatory affections, particularly ophthalmia and phthisis:" and lastly, that "for the most part, the measles, even when violent, are without any putrid tendency; but in some cases, such a tendency appears*." My copy of the printed work of Dr. Cullen has marginal notes, made by myself in his lecture-room, in 1780 and 1781, while an attendant upon his lectures; and the notes standing opposite to the above passages respectively, are as follow. "In the last year's course (of lectures) I find, that Dr. Cullen supposed the morbillous matter to have been determined to the mucous glands, (when the flux occurred.)" "If there is an acrid matter, he does not know how to evacuate (or correct) it; and therefore he attends to the inflammatory circumstances." "As he never saw this putrid tendency, he has always treated the measles as an inflammatory affection." It appears probable from a review of these three passages and notes, that Dr. Cullen had in his mind Dr. Huxham's account of a "violent mucous diarrhœa" seen in measles; and as to the putrid form of the measles, he cites for his authority, the celebrated paper of Dr. Watson above noticed †.

It is needless to insist here on the distinctions between the benign and malignant species of measles; though the small-pox, the dysentery, the scarlet fever attended with sore throat, and perhaps various other diseases, (even without including the venereal,) have each of them also two forms; which differ not only in degree, but in some respects in symptoms; according to constitution, season, treatment, infection, and other circumstances. Nor shall

 I pre-

---

album consisted of a decoction of the roots of tormentil and comfrey, to which were added cinnamon, calcined hartshorn, pure chalk, and white sugar. (See James.)

* See Dr. Cullen's first Lines of the Practice of Physic, Edition 1779, vol. 2, p. 80, 81. See also, several passages in his Materia Medica.

† See Medical Observations and Inquiries, vol. 4, p. 132.—In the same volume is Dr. Dickson's Vindication of Sydenham against Morton and Mead, on the subject of measles. Morton has certainly the bills of mortality and Sydenham's silence against him; but Morton does not speak upon hearsay, but upon the recollection of what happened many years before he wrote.

I pretend to detail the respective symptoms or treatment belonging to the two stages (answering to those in the small-pox,) which generally occur in severe, and often in the mild state of primary measles; for here common authors must be consulted, subject to what is above observed. But it is proper to notice, that measles even of the same species, under different circumstances, manifest considerable variety; which must not only govern the practice of the physician, but also his judgment of the practice of others. On the whole, one who prescribes for measles should never lose sight of what Sydenham says, after having spoken of small-pox. "If this be the true and exact history of the disease, (namely, small-pox,) he deserves to be blind, who will not see that the whole event of it, as to either part, depends on the foundation of the cure, well or ill laid at first."

This might seem the proper place to say a few words upon secondary diseases, as allied to measles. But I shall combine the trifle which I have to note on this subject, with some other corresponding articles, in the division of this letter which immediately follows.

### *Of connected or corresponding Diseases.*

These are of several descriptions.

I. We have already noticed certain disorders which frequently follow each other, by a fixed order in nature, in separate individuals; without relation to other patients, and independent in a great degree on the seasons. Measles we have seen accused, as being the frequent parents of diseases in the chest, eyes, and intestines. Small-pox is thought often to produce scrophula and other diseases; or (to use the words of Haller) their troublesome consequences may be ulcers, inflammation of the eyes, weakness of the joints, loosenesses, or tedious diseases of the lungs. These are the most eminent among the many examples of primary diseases leading to secondary ones. But secondary diseases may sometimes lead to a third class, or to ternary diseases; for obstruction or inflammation may occasion schirrus, and schirrus may end in open ulcer; and if the obstruction or inflammation in these cases has been the sequel of some prior acute disease, we shall then have four diseases in lineal descent; with three periods of apparent health perhaps interposed.

The several diseases resulting from measles, are not only like the primary complaint itself, commonly either acrid or inflammatory; but, in some cases, they are perhaps haps the consequence of re-action. The small-pox generates diseases of a more mixed order; but they also are commonly either acrid or inflammatory. Sydenham, speaking on this subject, says, "When the patient is upon the recovery, and the pustules are fallen off and he has eaten flesh a few days, (namely about the one and twentieth day,) I reckon he may be bled in the arm, if the disease has been violent; for the inflammation which the small-pox has impressed upon the blood (whether the patient be old or young,) no less indicates blood-letting, than the filth that has been gathered together does purging. This is evident enough, both from the colour of the blood that has been taken away after the small-pox has been severe, (which is like that of pleuritics;) and also from those great inflammations that fall on the eyes after this disease; as also from other ill effects of the blood overheated and depraved by this disease: which is the reason that they who were healthy all their lives before, do (that is many of them,) all their time after contend with sharp hot humours falling on the lungs or some other part. But if the pustules have been few, there will be no need of bleeding. After bleeding, I give three or four purges. Moreover, when the patient has been freed a while from the confluent small-pox, and rises daily, it sometimes so happens that he is cruelly troubled with a swelling of his legs; which either goes off of its own accord after bleeding and purging, or is easily assuaged by the use of discussing and emollient herbs boiled in milk."

II. Sydenham, and our own experience have made us acquainted with another form of connection between diseases; I mean that, by which two diseases of independent origin influence each other. Thus a departing epidemic acts upon a commencing one, and receives back its re-action; certain symptoms of each being modified by the other. An ordinary disease also has some of its characters changed by a cotemporary epidemic.

If it be said, that a certain state of the air influences in these cases, it must still be admitted that a past disease may have partially changed our bodies for a time; and that infection also, in spreading from one to another, may have limited effects upon certain individuals; which are concessions sufficient for our purpose.

III. One epidemic in some cases seems to go even to the length of generating others, making them partly cotemporary with itself, though perhaps in different sets of patients; or else the same state of the air or some other

 unknown unknown circumstance, has the power to produce related diseases.

Of this I shall here offer several examples.—"In the first two months (says Sydenham) that this sort of measles (in 1674) appeared, a measly fever here and there intervened, in which some pimples broke out in the trunk of the body (especially in the neck and shoulders) like the measles; but they were distinguished from them, because they did not seize the whole, being confined to those parts we now mentioned. But the fever, though it was plainly of the same kind, was more violent, and continued fourteen days and sometimes longer. It bore neither glysters nor bleeding, being enraged by both; but the method for the measles agreed with it." *

At Edinburgh, in 1735 and 1736, we learn, that "during the measly season, several people, who never had the measles, had all the preceding symptoms of measles; which went off in a few days without any eruption (which they underwent months or years afterwards.) Others, who had undergone the measles formerly, had at this time a fever of the erysipelatous kind, with eruptions like to what nettles cause; and all the concomitant symptoms of measles from the beginning to the end of the disease.†

What is stated as sometimes occurring in seasons when the measles prevail, has its parallel in seasons of the small-pox. I begin as before with Sydenham, who writes thus. "At the time (1667) wherein the small-pox first broke out, a new fever arose, not much unlike the small-pox; if you except the eruption of the pustules, and the symptoms which depend upon them. This fever did not seize near so many as the small-pox, yet it continued as long. But in the winter, when they decreased, this prevailed; and when they returned, this receded: yet did it never quite cease at these times; till at length in August, 1669, the small-pox and this fever went off together."—The agreement between this fever and the small-pox chiefly lay in a pain near the pit of the stomach, which manifested itself strongly on pressure; also in a natural salivation, and natural sweats; in spots; and in the mode of treatment being common to both. Hence, Sydenham called the fever variolous. Sydenham even informs us, that a third disease accompanied these two epidemics, especially during

* See Peachy's Translation of Sydenham. Sect. 5. c. 3. p. 165.

† See Medical Essays and Observations, 5. 28. 29.

during one summer, namely a looseness; the constitution of the air inclining to a bloody flux: and he adds, that it was manifest, that this disease was nothing else but the fever turned inwards upon the bowels. In other words, he supposed it owing to an inflammatory tendency conveyed, through the mesenteric arteries to the intestines; there "soliciting excretions." It was cured by bleeding, and the cool regimen; and became mortal (he says) from the use of astringents, and even of rhubarb and other gentle cathartic.

Perhaps it may be worth remembering here, that there are many instances where the infectious matter of the small-pox and kine-pox, applied to persons who have before passed through those diseases respectively, has been capable of producing a representation more or less perfect of certain of their symptoms.

IV. Another species of connection between diseases is seen, when disorders intermix with each other, in the same patient, and in the same moment of time, by a certain alliance. This may so happen, that one shall appear as the mere symptom of the other; as sore eyes, cough, and looseness seem to be symptoms of the measles, during the primary disease of measles. Syphilis and gonorrhœa also, if they be really distinct diseases, are in many cases found blended with each other in the same patient.

V. The last connection, or here I shall rather call it correspondence, which I shall mention between diseases, is, where one is vicarious with respect to another; that is, is of a nature to represent and perform the offices of the other. A more brilliant instance cannot be produced, than that of the substitution of the kine-pox for the small-pox. The kine-pox possesses all those secret influences upon our frame, by which it disables us from receiving the small-pox; in this, exactly imitating the small-pox itself, which almost universally shuts the door against its own return, whenever it retreats from its visitation of any individual; as if it had there destroyed the means and pabulum of its own existence on every future occasion.

And here let me be forgiven for expressing my admiration at the power of vaccination, (under our present notions at least of its efficacy,) in securing us from the small-pox in all its forms. Inoculation of the small-pox had already accomplished wonders of an inferior kind. It had turned the current of the small-pox from the confined vitals to the open skin; where the healing air has access to the parts which are most affected; where the ravages of the disease, if they do occur, are less important, than when falling upon the vitals; and where the matter of all the pustules has a ready issue.* But what vaccination proposes to do, is far beyond this; especially as it removes the mortality which inoculation itself spreads, in consequence of its extending the infection in a natural form. The prevention of one serious disease by the infliction of another mild one, which is of itself borrowed from another race of animals, (instead of our retaining that serious disease to multiply its fatal likeness, and to introduce new cases of calamity) is a singularity hitherto not even looked for in medicine. It may perhaps be considered as a peculiarity on this occasion, little less worthy of notice, that an attempt to extinguish a disease so lucrative to practitioners, as the small-pox, should have excited but little intrigue; that vanity also should have called up only a few opposers; that few real difficulties should have occurred to be solved respecting the progress or the management of the kine-pox; and that the knowledge of the inoculation for this new disease should have spread itself more rapidly over the globe, than any other human invention. Perhaps in the end, we may find, that it is scarcely to be called a human invention; and that men have only been used as instruments respecting it. In the mean time, we are allowed to hope, that it will go on to justify itself as one of the first improvements in medical practice, as well in statistical economy; at the same time that it offers a grateful safe-guard to our private feelings. It would indeed be no diminution of the effective value of this measure, should the small-pox itself be supposed heretofore to have had one and the same origin with the kine-pox; in short, to be only a more formidable species of kine-pox attended with contagion; which in our day, is thus exchanged for a gentler form of it. The wonder of the case may cease, but not the blessing. Perhaps so simple a representation of the transaction as this, if generally received, might even extend the blessing; by reconciling every individual to

* I do not pretend to say, that the pustules are generally found internally in small-pox; for I know to the contrary; and perhaps pustules may form one of the advantages of inoculation, especially as their discharge is outward. But if the remark of Haller be true, respecting the state of the intestines in the malignant form of small-pox; charcoal, oxygenated air, and other materials, may be given by the mouth and by injections, to meet the variolous matter in the intestines; and a farther proportion of oxygen may be made to enter the system through the lungs.

to a belief of the general truth in question, and of the practical advantages to be drawn from it. In the mean time I cannot but remark, that to the British nation and their descendants, are owing the introduction of the inoculation of the small-pox and the kine-pox; and the proper management of the two distempers.

I shall only remark farther on the subject of secondary diseases, that as so many of them are enlisted in the train of the small-pox; it may be wise, while our opportunities still last for making dissections of the dead in this disease, to pursue our observations on the subject. If the small-pox should finally yield to vaccination, the knowledge obtained may still be useful; as all discoveries into nature have commonly more than one application.

### *Of Inoculation for the Measles.*

The beautiful practice of inoculation for the small-pox, which was long employed in various parts of the east, before it was even known either in Europe or America, has been imitated for several other diseases in Europe; and, among the rest, for the measles.

It is now nearly half a century, since Dr. Francis Home of Edinburgh, proposed this measure for measles. Dr. Percival says, that it was "practised in several instances with considerable success; the soreness of the eyes being mitigated by it, the cough abated, and the fever rendered less severe. Dr. Home's method of communicating the infection was, by applying (to an incision in each arm) cotton moistened with the blood of a patient labouring under the measles. But the morbillous matter (adds Dr. Percival) has since been engrafted, by means of lint, wet with the tears which flow from the eyes in the first stage of this disorder. It is to be lamented (continues Dr. Percival, that so little attention has been paid to this valuable improvement of the healing art."* Dr. Underwood agrees, that report was favourable to what Dr. Home had done: and says that the practice seemed at one time to have been imitated in Sweden. Dr. Beddoes also appears to think well of it. But Dr. Underwood and Dr. Beddoes seem to admit, that it is now little attended to in Great Britain.†

One

---

* London Medical Observations and Inquiries, 5. 285-286, and Home's Medical Facts and Experiments.

† Underwood on the Diseases of Children. 4th Edition. I, 297, and Medical Abstracts. 4. 239. 4th Edition.

One reason, perhaps of this general neglect is, that measles are chiefly contemplated by some, as only dangerous in their primary form; that by others, the fury of the first disease has been easily quelled, and the occurrence of the secondary disease easily prevented; and that measles do not, like the small-pox, permanently deform the human countenance. If there are any stronger reasons than these, for the disuse of the practice, they ought to be made known by those who are possessed of them; otherwise the experiment ought to be resumed.

### *Of Dissections.*

The only solid observations made in this letter, having been founded upon dissections, it would be wrong to quit this subject without observing, that since the benefits to be derived from opening the bodies of those who die of singular diseases is commonly evident, the privilege of doing it should oftener be solicited. In our town, the indulgence is now seldom refused; as it is never sought but upon critical occasions, and is frequently attended with useful discoveries. When the surviving friends (in addition to the interest they take in the information obtained,) observe decorum, neatness, and a desire to prevent trouble, in the operator; one dissection commonly leads to another, even upon the suggestion of the friends of the deceased. This the more readily happens, as a few persons of consequence have led the way. Dissections have also in general the advantage of calming the friends of the deceased, as to the necessity of the death which has occurred; especially where the disease has been treated prudently, and by rule. A man of judgment at the same time will perceive, that the appearances in the dead body, however certainly they may point out the immediate cause of death, will have sometimes proceeded from the errors of the practitioner, as well as from the primary disease. This reflection will seldom embarrass a pretender; nor ought it to discourage a man anxious to improve, either himself, or his art, which is so necessary to mankind.

Before I quit this subject, I shall venture to hint, that a little treatise on the art of dissecting seems a desideratum in modern times. It is not merely necessary to learn how to prepare the head, the thorax, or the abdomen for dissection; which is all that is taught on this subject by Mr. Benjamin Bell. The young practitioner ought to know (what is not always taught even by anatomists;) I mean, the general size, form, weight, colour, consistence, and habit habit of each organ, in a state of health, according to age and to sex; and all this should appear within the compass of a small number of pages. The practitioner ought to be informed too what diseases are related to each other; what parts have concern in each disease; and under what form each disease operates upon each part. I have seen a dissector ignorant of the most simple things; when the proposed treatise, consulted even while he had the knife in his hand, might have informed him. Much may be learned in the examination of the dead, without delicate skill or profound knowledge; and physicians, apothecaries, and curious persons, possessed only of a slight knowledge of anatomy, might soon be qualified to perform many useful inspections of the dead. The knowledge of the seats and of the internal effects of diseases, would thus be perfecting itself, by means more extensive than any now employed; and the knife, instead of chiefly instructing the mere anatomist and surgeon, would enlighten the person, who after all, has most to do with the human body, namely, the physician.

One chapter ought to be devoted to diseased appearances; as aneurisms, hydatids, polypi, calculi of different kinds, tubercles, schirrus, and cancer. And with respect to cancer, I think the work of Dr. Adams on cancerous breasts deserves consultation, as exhibiting hints which require to be further examined.

Another chapter may be given to an enumeration of the points proper for the dissector to notice, in the written account of his dissections. And here Dr. Stark's mode of comparing the symptoms with the morbid appearances, is much to be commended; and Dr. Baillie likewise has done well in generalizing the accounts of the symptoms. If the method of treatment used could also be given, and given with accuracy and candour, it might safely be recommended.

A third chapter may detail the preparatory measures to be taken in every dissection; and they are very few.

A fourth may include the steps necessary to secure the operator from danger in critical cases. It has happened to me, as it doubtless has to you, to have had several of my acquaintance die or become dangerously ill, from the infection conveyed by the puncture of the dissecting knife, or the effluvia from a dead body. As I am not of the profession, but merely amicus curiæ, I may with propriety testify, that a zealous practitioner among the sick and dead, runs more hazards to his health and life, than he has has credit for; and has therefore to add these to his other claims for remunération. No professions, however, generally speaking, are worse rewarded than those of the medical practitioner, the clergyman, and the school-master, as far as depends on voluntary pay; so careless, in appearance, are we of our bodies and souls, and the improvement of our intellect.

If the plan above recommended were sensibly pursued, I think a few engravings would be added, consisting almost solely of outlines.* The parts might first be shewn in situ, and afterwards some of them might be given in different sections or layers; and some of them might be exhibited separately, as the intestines; (which might be shewn first in one extended line, with their membranous attachments; and then convoluted; with letters serving for reciprocal references between the two.) Indeed I may say here, that in order to aid surgical operations on the living, there are many cases where transverse and other sections of parts, as of the limbs, might very usefully be exhibited.

The two Hunters used to boast, that when they began their lectures in London, there were few towns in England, where practitioners resided, who were able to describe a case, or a dissection; but that as they proceeded, the number of these so increased, that there were few towns where one or more did not reside, capable of managing on these occasions in a respectable manner.

The same progress is now taking place in part of the United States; and the little work proposed, would considerably add to its rapidity.

We may be the more anxious, for some measures on these subjects, as by submitting select cases to dissection, we should perpetually obtain fresh knowledge. Upon this principle, I have myself seen no dissection from which I have not learned considerably; for, to use the remark of Sterne, an ounce of a man's own sense, is worth a ton of other people's.

Should any part of what has here been suggested or collected, have the good effect, my dear sir, of exciting you to apply your original and happy turn of thinking, and your extensive experience and reading, to the subjects to which

---

* If more than outlines be given, and colours be rejected, the parts may be distinguished from each other by the interchange of etching, scraping, dots, and lines in the copper-plates.

which it relates, I shall certainly not regret my trouble. Highly important, indeed, is the principal object of the inquiry; since you must readily agree, that besides the deaths from the primary state of measles, it is probable that many deaths arise out of the consequences of this disease, which are not placed to its account in the ordinary bills of mortality.

*District of Maine, March* 9, 1805.

